# Effectiveness of a brief hope intervention for chronic kidney disease patients on the decisional conflict and quality of life: a pilot randomized controlled trial

**DOI:** 10.1186/s12882-022-02830-7

**Published:** 2022-06-14

**Authors:** Kitty Chan, Frances Kam Yuet Wong, Suet Lai Tam, Ching Ping Kwok, Yuen Ping Fung, Ping Nam Wong

**Affiliations:** 1 School of Nursing, The Hong Kong Polytechnic University, Hung Hom, Kowloon Hong Kong SAR, People’s Republic of China; 2 Department of Medicine and Geriatrics, Kwong Wah Hospital, 25 Waterloo Road, Kowloon, Hong Kong SAR, People’s Republic of China

**Keywords:** Hope, Chronic kidney disease, Decisional conflict, Quality of life, Palliative care, Randomised controlled trials

## Abstract

**Background:**

Stage 5 chronic kidney disease (CKD) patients often experience decisional conflict when faced with the selection between the initiation of dialysis and conservative care. The study examined the effects of a brief hope intervention (BHI) on the levels of hope, decisional conflict and the quality of life for stage 5 CKD patients.

**Methods:**

This is a single-blinded, randomized controlled trial (ClinicalTrials.gov identifier: NCT03378700). Eligible patients were recruited from the outpatient department renal clinic of a regional hospital. They were randomly assigned to either the intervention or the control group (intervention: *n* = 35; control: *n* = 37). All participants underwent a customized pre-dialysis education class, while the intervention group received also BHI. Data were collected prior to the intervention, immediately afterwards, and one month following the intervention. The Generalized Estimating Equation was used to measure the effects in the level of hope, decisional conflict scores (DCS) and Kidney Disease Quality of life (KDQOL-36) scores. Estimated marginal means and standard errors with 95% confidence intervals of these scores were also reported to examine the within group and between group changes.

**Results:**

An increase of the hope score was found from time 1 (29.7, 1.64) to time 3 (34.4, 1.27) in the intervention group. The intervention had a significant effect on the KDQOL-36 sub-scores Mental Component Summary (MCS) (Wald χ^2^ = 6.763, *P* = 0.009) and effects of kidney disease (Wald χ^2^ = 3.617, *P* = 0.004). There was a reduction in decisional conflict in both arms on the DCS total score (Wald χ^2^ = 7.885, *P* = 0.005), but the reduction was significantly greater in the control group (effect size 0.64).

**Conclusions:**

The BHI appeared to increase the level of hope within the intervention arm. Nonetheless, differences across the intervention and control arms were not significant. The KDQOL-36 sub-scores on MCS and Effects of kidney disease were found to have increased and be higher in the intervention group. The DCS total score also showed that hope was associated with reducing decisional conflict.

**Trial Registration:**

ClinicalTrials.gov Protocol Registration, NCT03378700. Registered July 12 2017.

**Supplementary Information:**

The online version contains supplementary material available at 10.1186/s12882-022-02830-7.

## Background

With the rising prevalence of end stage kidney disease (ESKD) around the world, an increasing number of patients who have been diagnosed with stage 5 chronic kidney diseases (CKD) must decide whether to undergo renal replacement therapies (RRT) [1]. Anxiety, ambivalence [2], and decisional regret [3] are common reactions when patients are faced with making a choice between undergoing a conservative treatment or initiating RRT. Regardless of the benefits in terms of economic costs, mortality risks, and quality of life (QoL) from initiating dialysis in stage 5 of CKD [4], many such patients opted for conservative management or palliative care [5–7].

In a large study, it was pointed out that early initiation of RRT should not be based on the estimation of glomerular filtration rate only (estimated glomerular filtration rate [eGFR] of 10–14 mL/min/1.73 m^2^) [8]. Prognostic uncertainty and the subjective perception that their health could worsen after receiving RRT are factors affecting the decision that patients make [9]. Some patients felt unprepared or uninformed about their treatment options because of a lack of robust CKD decision-support interventions to thoroughly discuss the conservative management of CKD [3]. Others felt pressured to make a decision, and often felt decisional regret when their condition deteriorated [10]. It is noteworthy that a sense of hopelessness was documented in patients with chronic kidney diseases [11–14]. Therefore, standard pre-dialysis education, which involves providing information on the comparative risks of different forms of treatment, is insufficient to guide individual decisions that are affected by personal values and perceptions [15].

To help patients make decisions that best fit their own situation, it was suggested that a proactive cognitive and emotional psychotherapeutic intervention be incorporated in an educational model [2]. The cognitive appraisal of patients with ESKD, referring to the perception of their coping abilities and their belief that their condition can be controlled, has a large effect on their levels of depression and stress [16]. Hope is a target-oriented cognitive process that comes into play when appraising one’s capacity to reach set personal goals [17], propose new solutions, and implement actions in a flexible, creative way [18]. Hope made the patients more engaged in making decisions about conservative care [19]. It was also found to heighten the positive expectations of patients [20–24] and to influence their attitudes and actions towards persevering to achieve their chosen goals [25, 26]. Furthermore, a study has shown that after the implementation of a hope intervention, the resulting higher levels of hope were associated with reduced physical and psychological symptoms [27]. In a meta-analysis, hope was found to be one of the strongest factors in psychotherapy leading to positive change [28].

A nurse-led low-intensity psychoeducational hope intervention was initiated in this study with an aim to assess the change in the hope level in patients with stage 5 CKD, who received the hope intervention. In addition, we explored whether the participants experienced any change in decisional conflict and in their QoL as a result of receiving the hope intervention.

## Methods

This study was a single-blinded, two-arm randomized controlled trial (ClinicalTrials.gov identifier: NCT03378700) and adhered to the CONSORT guideline [29]. The study protocol was published elsewhere [30]. Only the principal investigator and the nurse who delivered the intervention were aware of the patients’ group allocation. Allocation concealment was applied when recruiting participants from a telephone list of those eligible to take part in the study, using a random numbers table. Patients’ baseline and endpoint surveys were administered by an independent research assistant, who was not involved in the education or intervention programmes. Participants in both the intervention and control groups received 1 h of pre-dialysis education led by renal nurse clinicians, who offered advice on treatment modalities. They also received the standard patient care, which included a follow-up in an out-patient clinic to monitor the course of their illness and receive necessary treatment. Afterwards, the control group received social telephone calls once a week for two weeks, while the intervention group received the 4-week Brief Hope Intervention (BHI).

### Study setting and participants

All stage 5 CKD patients (glomerular filtration rate (GFR) ≤ 15 mL/min/1.73 m^2^) attending the outpatient renal clinic of a regional Hong Kong hospital and who were planning to initiate RRT or conservative care were recruited for the study. The criteria for inclusion were: age ≥ 18 years; alert and oriented; able to speak Cantonese; able to read and write Chinese; having no hearing deficit; and reachable by mobile phone [30]. The study was approved by the research committees of the university (Reference number HSEARS20180601002) and the regional hospital in Hong Kong (Reference number KC/KE-18–0018/ER2) with which the research members are affiliated.

### Sample size

Sample size was calculated based on changes in the effect size of the hope scores of the participants [30]. Assuming a two-tailed correlation alpha value of 0.6 (significance level at 0.05) to allow for a detection of 0.5 in effect size with a power of 0.70 [31] and adjusting for attrition, the required sample size of each group was 36 [32, 33].

#### Brief hope intervention

Brief Hope Intervention (BHI) is a low-intensity psychoeducational approach that could help CKD patients establish feasible goals when faced with the decision of whether or not to initiate RRT. A theory of hope was adopted [22, 25] to guide the key strategies used in the intervention: (1) goal setting (goals), (2) problem-solving (pathways), and (3) positive self-talk (agency) [17]. Hope motivates people to pursue goals with a positive outlook, thereby promoting meaning in life and personal strengths [34]. The brief hope intervention (BHI) began with a pre-dialysis education class (see the pre-dialysis education programme for details), followed immediately by the Brief Hope Intervention (BHI) [35]. The BHI consisted of four sessions held on a weekly basis: two 1-h face-to-face sessions and two 30-min telephone follow-up sessions in between. The programme was validated by experienced renal care clinicians and academic staff. In each session, the participants were coached to make well-thought-out decisions based on the hope framework. (1) Goal setting – establishing feasible goals in response to the treatment options: dialysis initiation or conservative care, (2) pathway thoughts – gathering relevant facts related to available options, performing hope visualization exercises to solve problems when pursuing set goals, (3) agency – revising the goal that was set and affirming the choice of treatment through positive self-talk. Fidelity to the intervention was achieved by adhering to the manual for the programme and delivered by the same nurse, who was experienced in offering the BHI [30]. Please refer to the supplementary file for an example of goal setting, visualisation and positive self-talk in the Brief Hope Intervention.

### Pre-dialysis education programme

This was a customised one hour educational class offered by the renal nurse specialists. The objective was to provide information on the various treatment modalities available for ESRD patients. The nurse specialist would help the patients to know what to expect when a RRT was initiated or before a conservative treatment. The concept of palliative care was introduced when the selected treatment became ineffective. In addition, in the second and the third week after the educational class, the patients received social communication phone calls initiated by a trained social worker.

### Data collection

**Fig. 1:**
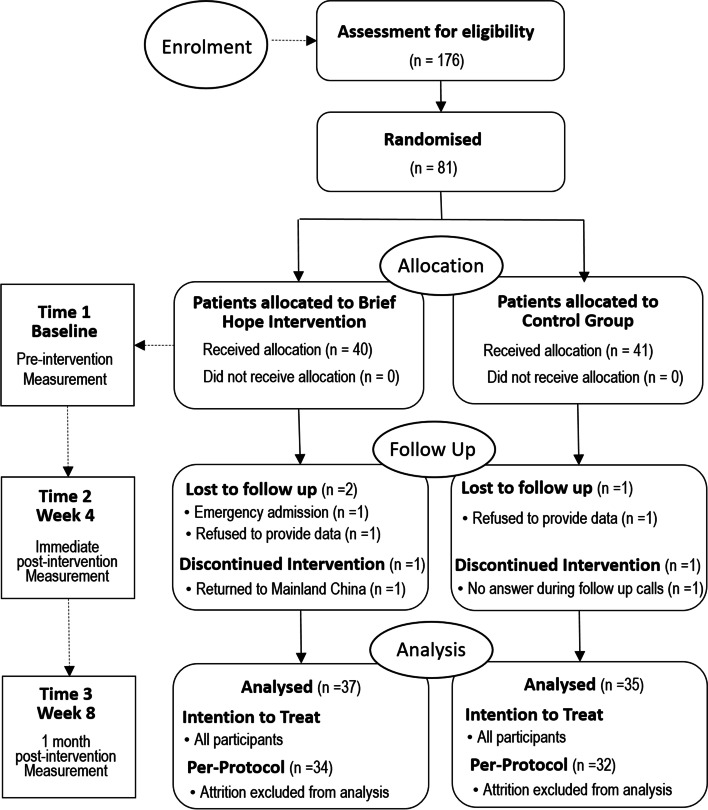
Consolidated Standards of Reporting Trial (CONSORT) flow chart of the randomized controlled

Data were collected at three time points. Time 1 (T1) – immediately before the commencement of the pre-dialysis education programme. Time 2 (T2) – immediately post-intervention, meaning after the completion of BHI in the experimental group, or the social calls in the control group. Time 3 (T3) – four weeks after the completion of the programme. Figure 1 presents the flow in the collection of data, using the Consolidated Standards of Reporting Trials (CONSORT) [29].

### Outcome measures

The primary outcome was the change in level of hope toward ongoing or future events in the individuals’ lives [36], as measured using the 6-item State Hope Scale (SHS). The scale consists of two subscales, namely on (1) agency and (2) pathway of hopeful thinking [37], rated on an 8-point scale with 1 = definitely false and 8 = definitely true. Higher sum scores indicate a higher hope level. The instruments were reliable, with Cronbach’s alphas of the SHS ranging from 0.74 to 0.93, and the comparative fit index was 0.94 [37].

Decisional conflicts and QoL were the secondary outcomes. The 16-item Decisional Conflict Scale (DCS), rated on a 5-point Likert scale, measured the patients’ decisional conflict when choosing between RRT and conservative care [38, 39]. The DCS consists of five subscales: (1) uncertainty, (2) being informed, (3) values clarity, (4) support, and (5) effective decision. The total and subscale scores were transformed, ranging from 0 to 100, with higher scores indicating more decisional conflict. Higher scores indicate more conflict in decision making. The root mean square error of approximation (RMSEA) was 0.63, while the test–retest reliability alpha value exceeded 0.78 [40]. It was also found that for every unit increase in the DCS, participants were five times more likely to express decisional regret.

The Chinese version of the Kidney Disease Quality of Life Questionnaire (KDQOL-36) [41] was used to measure QoL. The higher scores indicate better QoL. It consists of 24 items with four subscales on (1) symptoms and problems, (2) burden of kidney disease, (3) effects of kidney disease, and (4) a Short Form Health Survey (SF12) – consisting of two subscales on physical component summary KDQOL-36 (PCS) and mental component summary KDQOL-36 (MCS). The RMSEA was 0.63 and the alphas of the five subscales ranged from 0.76 to 0.92 (*p* < 0.001). A high intraclass correlation of > 0.98 was reported in the test–retest reliability.

Gender, age, marital status, educational level, occupation, and income were reported. The patients’ medical diagnosis and GFR were retrieved from their clinical records. The approximate time to complete the entire survey was approximately 15 min.

### Data analysis

Data analyses were performed using SPSS 24.0. Descriptive statistics were used to summarize the characteristics of the participants: frequency distribution for categorical data, means and standard deviations for continuous data. Characteristics of the participants, such as their demographic profiles, health-related variables, and outcome measures, were compared between groups at baseline (T1). A chi-square test and Fisher's Exact test were used to examine the difference between the groups in terms of the categorical variables, while Student’s T-test was used for the continuous variables. Intention to treat (ITT) and per protocol (PP) analyses were conducted [42]. PP was defined as participants attending both face-to-face sessions and at least one telephone follow-up session. This will be considered the threshold for receiving the minimum dose of the intervention.

The Generalized Estimating Equation (GEE) was used to measure the differences or changes between the intervention and control groups (between-group effects), as well as the changes at each time interval (immediately after the intervention (T2) and four weeks after the intervention (T3) with respect to its baseline within group (time) and interaction (group x time) [43]. A linear link function was used for continuous outcomes, namely, SHS, DCS, and KDQOL-36. A binomial link function was employed for dichotomous data such as health service utilization outcome. Correlations between hope and decision outcomes were also measured using GEE. All models were adjusted for age and sex. Estimated marginal means (EM means), standard error (SE), absolute differences with 95% confidence interval (CI) were reported at each time point. A two-sided level of significance was set at *p* < 0.05. Cohen’s d was used to calculate the effect sizes between the intervention and control groups, and within groups at time 2 and time 3. These scores allowed further scrutiny of the effect of health outcomes, which may contribute to capture the minimal clinically important difference (MCID) from a distribution-based approach in future studies [44, 45].

## Results

### Baseline characteristics

The study period was between September 2018 and November 2019. Of the 176 stage 5 CKD patients, 116 were eligible to take part in the study and 72 agreed to do so, for a response rate of 62.1%. The major reasons for refusing to participate were problems with mobility or transport to the hospital, currently residing in mainland China instead of Hong Kong, a lack of time, or an unwillingness to receive renal care or related information. The mean age of the participants was 66.7, with a standard deviation of 11.9. Forty-two (58.3%) of the participants were male and 30 (41.7%) were female. About two-thirds were married or cohabiting, and 59.8% had attained a secondary level of education or higher. On average, they had two diseases, with hypertension (76.4%) and diabetes (44.4%) being the two most common chronic diseases. Comparing the sample profile (Table 1) and baseline outcome measures (Table 2) of the intervention (*n* = 35) and control groups (*n* = 37), no significant differences in demographic and outcome measures were found between the groups. There were no significant differences in the GEE results of the ITT and PP groups. Therefore, only the ITT results are reported. Four weeks after the intervention, five participants from the control group were still indecisive about their treatment option, while three participants in the intervention group were indecisive. Moreover, four of the participants from the control group and 13 from the intervention group selected conservative treatment.

**Table 1:** Demographic characteristics of the participants at baseline

	Control (*N* = 37)	Experimental (*N* = 35)	Total	
	*N* %	*N* %	*N* %	*P*
Age (mean ± sd)	65.0 ± 11.0	68.4 ± 8.7	66.7 ± 11.9	0.231^c^
Gender				0.780^b^
Male	21 (56.8)	21 (60.0)	42 (58.3)	
Female	16 (43.2)	14 (40.0)	30 (41.7)	
Marital status				0.373^a^
Single	4 (10.8)	8 (22.9)	12 (16.7)	
Married/cohabiting	29 (78.4)	21 (60.0)	50 (69.4)	
Separated/divorced	2 (5.4)	2 (5.7)	4 (5.6)	
Widowed	2 (5.4)	4 (11.4)	6 (8.3)	
Educational attainment				0.745^a^
No formal education	5 (13.5)	5 (14.3)	10 (13.9)	
Primary	8 (21.6)	11 (31.4)	19 (26.4)	
Secondary	22 (59.5)	18 (51.4)	40 (55.6)	
Postsecondary/tertiary	2 (5.4)	1 (2.9)	3 (4.2)	
No. of diseases				0.700^a^
0	5 (13.5)	3 (8.6)	8 (11.1)	
1	10 (27.0)	8 (22.9)	18 (25.0)	
2	13 (35.1)	12 (34.3)	25 (34.7)	
3	7 (18.9)	6 (17.1)	13 (18.1)	
4 or more	2 (5.4)	6 (17.1)	8 (11.1)	
Heart disease				
Yes	5 (13.5)	6 (17.1)	9 (12.5)	
Hypertension				0.483^b^
Yes	27 (73.0)	28 (80.0)	55 (76.4)	
Diabetes				0.246^b^
Yes	14 (37.8)	18 (51.4)	32 (44.4)	
Cataract				0.430^a^
Yes	5 (13.5)	2 (5.7)	7 (9.7)	
Arthritis				0.900^b^
Yes	8 (21.6)	8 (22.9)	16 (22.2)	
Perceived current quality of life				0.140^a^
Very poor	0 (0.0)	0 (0.0)	0 (0.0)	
Poor	3 (8.1)	2 (5.7)	5 (6.9)	
Neutral	22 (59.5)	14 (40.0)	36 (50.0)	
Good	11 (29.7)	13 (37.1)	24 (33.3)	
Very good	1 (2.7)	6 (17.1)	7 (9.7)	
Perceived current health condition				0.899^a^
Very poor	0 (0.0)	1 (2.9)	1 (1.4)	
Poor	9 (24.3)	9 (25.7)	18 (25.0)	
Neutral	18 (48.6)	14 (40.0)	32 (44.4)	
Good	9 (24.3)	10 (28.6)	19 (26.4)	
Very good	1 (2.7)	1 (2.9)	2 (2.8)	

^a^Fisher's Exact test

^b^χ.^2^ test

^c^T-test

**Table 2:** Descriptive statistics of outcome measures at baseline

	Control (*N* = 37)			Experimental (*N* = 35)		Total		
	Mean		SD	Mean	SD	Mean	SD	P
State Hope Scale								
Total	29.2		8.45	29.8	9.47	29.51	8.91	0.767
Pathway	13.7		4.91	14.3	5.48	13.99	5.17	0.601
Agency	15.5		4.85	15.5	5.07	15.52	4.92	0.991
Decisional Conflict Scale								
Total	49.6		18.42	44.0	20.60	46.9	19.58	0.227
Being informed	62.6		30.97	56.4	31.77	59.6	31.30	0.406
Values clarity	65.5		32.22	52.4	29.12	59.1	31.25	0.074
Support	32.7		22.51	36.2	17.96	34.4	20.36	0.466
Uncertainty	55.4		26.15	49.3	31.20	52.4	28.68	0.369
Effective decision	36.5		24.09	30.2	18.28	33.4	21.55	0.217
Kidney Disease Quality of Life (KDQOL-36)								
Short form health survey (SF-12)								
KDQOL-36 (PCS)^a^	64.8		23.32	62.9	26.74	63.9	24.89	0.738
KDQOL-36 (MCS)^b^	74.3		23.90	67.9	24.98	71.2	24.48	0.266
Burden of kidney disease	54.2		31.39	49.5	26.79	51.9	29.14	0.492
Symptoms and problems	90.4		15.24	84.4	15.54	87.5	15.57	0.106
Effects of kidney disease	87.8		15.73	85.3	15.00	86.6	15.32	0.485

^a^Physical Component Summary

^b^Mental Component Summary

### State Hope Scale (SHS)

Regarding the total SHS scores in the GEE results (Table 3), no significant differences were detected in the effects of time (β = -0.659, 95% CI = (-0.824, 2.143), Wald χ^2^ = 0.759, *P* = 0.384), between groups (β = -1.383, 95% CI = (-6.703, 3.937), Wald χ^2^ = 0.260, *P* = 0.610), or in group-time interaction (β = -1.609, 95% CI = (-0.222, 3.603), Wald χ^2^ = 3.001, *P* = 0.083). However, a within-group change was noted in the intervention group from Time 1 to Time 3 (effect size 0.54), with estimated marginal means and standard errors increasing from T1: 29.7 (1.64), T2: 30.5 (1.42), to T3: 34.4 (1.27), but no significant difference was found in the control group (Table 4).

**Table 3:** Results of the GEE models

Variables		Adjusted Model^#^				
		β	SE	95% CI	Wald χ^2^	*P*
State Hope Scale—Total	Time	0.659	0.757	[-0.824, 2.143]	0.759	0.384
	Group	-1.383	2.715	[-6.703, 3.937]	0.260	0.610
	Group*Time	1.690	0.976	[-0.222, 3.603]	3.001	0.083
Decisional Conflict Scale—Total	Time	-16.911	1.419	[-19.692, -14.131]	142.126	< 0.001*
	Group	-10.551	5.629	[-21.584, 0.482]	3.513	0.061
	Group*Time	5.519	1.965	[1.667, 9.370]	7.885	0.005*
Decisional Conflict Scale—Being informed	Time	-24.705	2.731	[-30.057, -19.354]	81.863	< 0.001*
	Group	-16.551	9.735	[-35.632, 2.530]	2.890	0.089
	Group*Time	9.724	3.660	[2.550, 16.898]	7.058	0.008*
Decisional Conflict Scale—Values clarity	Time	-25.039	3.143	[-31.200, -18.878]	63.452	0.000*
	Group	-26.314	9.898	[-45.713, -6.915]	7.068	0.008*
	Group*Time	14.237	4.072	[6.256, 22.218]	12.225	< 0.001*
KDQOL-36 Subscales						
KDQOL-36—SF12						
KDQOL-36 (PCS)^a^	Time	1.873	2.041	[-2.127, 5.874]	0.843	0.359
	Group	-4.677	7.828	[-20.020, 10.666]	0.357	0.550
	Group*Time	3.590	3.328	[-2.934, 10.113]	1.163	0.281
KDQOL-36 (MCS)^b^	Time	-0.155	1.549	[-2.882, 3.192]	0.010	0.920
	Group	-12.575	7.096	[-26.483, 1.333]	3.140	0.076
	Group*Time	6.093	2.343	[1.501, 6.763]	6.763	0.009*
KDQOL-36—Effects of kidney disease	Time	-1.470	1.201	[-3.823, 0.883]	1.499	0.221
	Group	-7.111	4.148	[-15.241, 1.020]	2.938	0.087
	Group*Time	4.475	1.551	[1.435, 7.516]	8.324	0.004*

*KDQOL* Kidney Disease Quality of Life Questionnaire, ^a^Physical Component Summary; ^b^Mental Component Summary

^#^Age and sex adjusted; *SE* standard error, *CI* confidence interval

^*^p < 0.05

Only total scores and significant subscales scores of the respective scale are stated in this table. Full set of GEE results can be found in the supplementary table 1

**Table 4:**
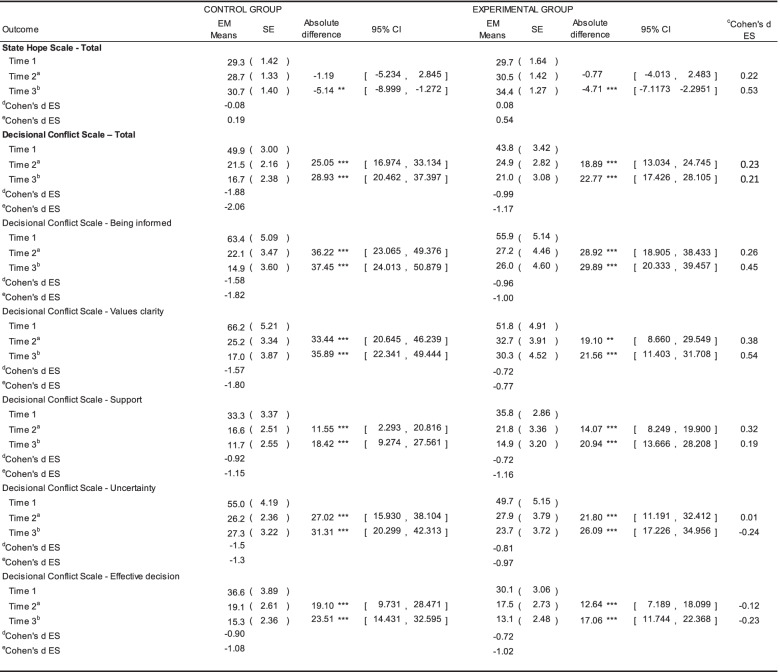
Results of the effect sizes between the control group and the experimental group, and the effect sizes within group across the three time points

*KDQOL* Kidney Disease Quality of Life Questionnaire, *EM Means* Estimated Marginal Means, *SE* standard error, *CI* confidence interval, *ES* effect size

^a^Absolute difference and significance level at time 2; ^b^Absolute difference and significance level at time 3; ^c^Cohen's d: effect size between control and experimental group; ^d^Cohen's d within group at Time 2; ^e^Cohen's d at Time 3

^*^p-value < 0.05; **P-value < 0.01; ***P-value < 0.001

Only total State Hope Scale scores, total and subscales scores of Decisional Conflict Scale are stated in this table. Full set of Cohen’s d results can be found in the supplementary table 2

### Decisional Conflict Scale (DCS)

Significant between-group differences were found in the total score and in two of the five subscale scores, namely ‘information’ and ‘value clarity’, but not in ‘support’, ‘uncertainty’, or in the question of ‘How effective or satisfied are you in making your decision?’ Total DCS in the control group were T1: 49.9 (3.00), T2: 21.5 (2.16) and T3: 16.7 (2.83), and were T1: 43.8 (3.42), T2: 24.9 (2.82) and T3: 21.0 (3.08) in the intervention group (Table 4 and Supplementary Table 2). The subscale scores of information in the control group were T1: 63.4 (5.09), T2: 22.1 (3.47) and T3: 14.9 (3.60), and in the intervention group were T1: 55.9 (5.14), T2: 27.2 (4.46) and T3: 26.0 (4.60), respectively. The subscale scores of value clarity were T1: 66.2 (5.21), T2: 25.2 (3.34) and T3: 17.0 (3.87) in the control group, and T1: 51.8 (4.91), T3: 32.7 (3.91) and T3: 30.3 (4.52) in the intervention group. Statistically significant time effects (β = -16.911, 95% CI = (-19.692, -14.331), Wald χ^2^ = 142.126, *P* < 0.001) and group-time interaction effects (β = 5.519, 95% CI = (1.667, 9.370), Wald χ^2^ = 7.885, *P* = 0.005) were found in the DCS total score. When compared with the baseline, the mean DCS total score at T2 decreased by 28.4 and by a further 4.8 at T3 in the control group, while the corresponding figures for the intervention group were a decrease of 2.9 and 3.9, respectively. Regarding the subscales of DCS, DCS-Being informed had the same effects as the total score, with significant time effects (β = -24.705, 95% CI = (-30.057, -19.354), Wald χ^2^ = 81.863, *P* < 0.001) and group-time interaction effects (β = 9.724, 95% CI = (2.550, 16.898), Wald χ^2^ = 7.058, *P* = 0.008), respectively. The GEE model showed that in DCS-Values clarity scores, there were significant differences in the between-group (β = -26.314, 95% CI = (-45.713, -6.915), Wald χ^2^ = 7.068, *P* = 0.008), time (β = -25.039, 95% CI = (-31.200, -18.878), Wald χ^2^ = 63.452, *P* < 0.001), and group-time interaction effects (β = 14.237, 95% CI = (6.256, 22.218), Wald χ^2^ = 12.225, *P* < 0.001). When compared with the baseline, the mean DCS-Values clarity scores at T2 and T3 deceased by 41.0 and 8.2 in the control group, while the corresponding decrease in scores in the intervention group were 19.1 and 2.4, respectively. The results also showed that only significant time effects were found in DCS-Support (β = -10.997, 95% CI = (-14.391, -7.603), Wald χ^2^ = 40.327, *P* < 0.001), DCS-Uncertainty (β = -14.242, 95% CI = (-18.854, -9.630), Wald χ^2^ = 36.637, *P* < 0.001), and DCS-Effective decision (β = -10.854, 95% CI = (-14.455, -7.252), Wald χ^2^ = 34.891, *P* < 0.001) (Supplementary Table 1).

### Kidney Disease Quality of Life Questionnaire (KDQOL-36)

A significantly greater improvement in QoL was observed in participants in the intervention group T1. Regarding the subscales of KDQOL, statistically significant on group-time interaction effects were found for KDQOL-36 (MCS) (β = -1.549, 95% CI = (-2.882, 3.192), Wald χ^2^ = 6.763, *P* = 0.009), and KDQOL-Effects of kidney disease (β = -2.811, 95% CI = (-6.008, 0.385), Wald χ^2^ = 3.617, *P* = 0.004). No statistically significant between-group, time, or group-time interaction effects were found for KDQOL-Burden of kidney disease and KDQOL-Symptoms and problems (Table 3).

### Correlation of hope and decisional conflict

Hope was shown to be correlated with decisional conflict. When decisional conflict was set as the dependent variable, and time, group, and SHS were set as the independent variables, controlling for age and sex it was found that the higher the total score of the SHS, the less the decisional conflict. An increase of one unit in the total score of the SHS tended to lead to a reduction of 0.466 in the total score for decisional conflict (95% CI = (-0.752, -0.181), Wald χ^2^ = 10.329, *P* < 0.001), (Supplementary table 3). A significant reduction was seen in the DCS score of the participants in both groups, in contrast to the expected effect of the BHI, decisional conflict in the control group was significantly lower than that in the intervention group, with large effect size of 0.64.

## Discussion

This study began by examining the effectiveness of a novel, nurse-led intervention based on the hope theory to minimize decisional conflict and improve QoL when faced with treatment alternatives at stage 5 CKD. Although there was no significant group-time interaction effect in the level of hope between the intervention and the control group, significant changes in the sum scores and pathway scores in the level of hope was reported before and after the BHI within the intervention group. Pathway scores indicated hopeful thinking and problem solving abilities [37]. This aligns with the intervention strategies where the participants were able to carefully weigh their goals and the trade-offs with regard to the burden of RRT and conservative care. In the present study, hope was found to associate with decisional conflict and quality of life. Further investigation on how the scores meaningfully affect the patient outcomes within the MCID concept will throw further insights on the effectiveness of BHI.

It was found that the QoL increased significantly in the intervention group but not in the control group. The patients suffering from CKD might prefer and prioritise interventions that could potentially improve their QoL because it is a chronic disease [46]. The present results showed that an increase in level of hope was associated with improved QoL and less decisional conflict. Consistent with previous studies, where hope is considered a significant element in resolving conflicts because it is associated with the reframing of thought patterns [47]. In addition, hope was found to strengthen the motivation of patients to circumvent barriers to achieving lifestyle changes and to maintain healthy behaviours [48, 49]. Individuals with high levels of hope have been shown to be able identify and implement the best alternatives [50]. The present study suggests that adherence to the ethos of QoL for patients with stage 5 CKD needs to be supported not only through education and CKD-specific resources [51], but also with the inclusion of the element of hope.

Decisional conflict decreased significantly in both groups. It was interesting to find that a lower level of decisional conflict was observed in the control group than in the intervention group. Although setting goals and exploring a wider scope of alternatives are important steps in revealing conflicts and helping patients become accountable for the choice that they make, it may not dispel uncertainty. Significantly higher conflicts on the sub-scores for decisional conflicts: information and role clarifications, were observed in the intervention group than in the control group. These two components concern such aspects as the benefits of each option, the risks and side effects of each option, and the option that matters most to a participant. It is consistent with previous research where CKD patients might want to partner with their health care provider to discuss the possibility of delaying the initiation of dialysis for as long as possible [52] instead of making a firm decision. Instead of exercising autonomy, patients avoid seeking out and assessing information on their condition, but depend on physicians [53–55] or renal staff to provide information on valid treatment options [56]. The dilemma arises from the need to choose between aggressive treatment and conservative care to gain survival time or a better QoL as the major endpoint of therapy [57].

Furthermore, it has been suggested that some older adults may be less likely to engage in decision making and may prefer to select from fewer options, and be influenced by professional support [58–60]. This is consistent with the participant profile in the present study. Almost 60% of the participants in the present study were over 65 years old, with the eldest being a 92 year-old male. Perceived caregiver burden was significantly higher in older adults who selected RRT than in those who selected conservative care because of the intense care and support demands of RRT [61]. Given the high prevalence of CKD in elderly patients [62, 63], health professionals should be alert to the factors associated with decision making to facilitate communication and improve the capacity for patient and family involvement.

Taken together, amidst realistic concerns or dilemmas over selecting the most desirable treatment option, greater hope led to improved QoL, although it may not have had more of an effect than pre-dialysis education in reducing decisional conflicts. Regulating levels of hope is a starting point to developing interventions that promote problem-solving behaviours in relation to a conflict-related event [64].

### Limitations

While this was a randomized controlled trial, neither the participants nor the nurse clinician were blinded to the allocation. However, bias was minimized through allocation concealment, as the clinician was not involved in the randomization and allocation process. The present study was a single-centre trial and the results might not be generalizable to all stage 5 CKD patients. Moreover, the sample size of this study was small and the trial was underpowered to demonstrate an effect. There were increases in the hope scores over time in the BHI group when compared with the control group, however, these increases were not statistically significant. Although absolute differences and 95% CI was reported to reflect possible MCID and a potential efficacy of this novel intervention, it did not allow a robust conclusion in the treatment effect. Most patients with ESKD will be older adults, thus there will be some variability in their cognitive functions, co-morbidities, mobility, living arrangements, and socioeconomic support limitations. These conditions may restrict their motivation to set goals during the intervention, thereby affecting the health outcomes. The fidelity of the BHI could be further improved through evaluation of its recorded sessions.

## Conclusions

The results added foreground information on the contribution of hope to decisional conflicts. This was the first randomized controlled trial to address how to enable CKD patients to select the treatment options compatible with their personal needs, and to promote better health outcomes. In this pilot RCT, the BHI did not create a significant group-time effect on the level of hope, although the hope scores appeared to increase in the intervention group. The sub-scores on KDQOL-MCS and KDQOL-Effects of kidney disease were found to have increased and be higher in the intervention group than in the control group, with the former experiencing a significant improvement in their quality of life. We expect that CKD patients will construct their own trajectories in the decision-making process when faced with treatment options.

## Supplementary Information

**Additional file 1: Supplementary Table 1.** Results of the GEE models. **Supplementary Table 2.** Changes in level of hope, decisional conflict and quality of life at 4- (Time 2), 8- (Time 3) weeks after the intervention. **Supplementary Table 3.** Regression coefficient estimates of Hope on Decisional Conflict.

## Data Availability

The data sets used and analysed during the current study are available from the corresponding author on reasonable request.

## Abbreviations

ESKD: End Stage Kidney Disease
CKD: Chronic Kidney Disease
QoL: Quality of life
RRT: Renal replacement therapies
eGFR: Estimated glomerular filtration rate
GFR: Glomerular filtration rate
BHI: Brief Hope Intervention
SHS: State Hope Scale
DCS: Decisional Conflict Scale
KDQOL-36: Kidney Disease Quality of Life Questionnaire
