# Supplementary material for: Effectiveness of a brief hope intervention for chronic kidney disease patients on the decisional conflict and quality of life: a pilot randomized controlled trial
Source: BMC Nephrol. 2022 Jun 14;23:209. doi: 10.1186/s12882-022-02830-7 (PMC9195369; doi:10.1186/s12882-022-02830-7)
Supplement: Supplementary file 1 — Additional file 1: Supplementary Table 1. Results of the GEE models. Supplementary Table 2. Changes in level of hope, decisional conflict and quality of life at 4- (Time 2), 8- (Time 3) weeks after the intervention. Supplementary Table 3. Regression coefficient estimates of Hope on Decisional Conflict. [file 12882_2022_2830_MOESM1_ESM.docx]

Supplementary Table 1. Results of the GEE models.

| Variables |  | Adjusted Model | | | | | | | | | |
| --- | --- | --- | --- | --- | --- | --- | --- | --- | --- | --- | --- |
|  |  | β | SE |  | 95% CI | | |  | Wald χ^2^ | *P* |  |
| State Hope Scale - Total |  |  |  |  |  |  |  |  |  |  |  |
|  | Time | 0.659 | 0.757 | [ | -0.824 | , | 2.143 | ] | 0.759 | 0.384 |  |
|  | Group | -1.383 | 2.715 | [ | -6.703 | , | 3.937 | ] | 0.260 | 0.610 |  |
|  | Group*Time | 1.690 | 0.976 | [ | -0.222 | , | 3.603 | ] | 3.001 | 0.083 |  |
| State Hope Scale - Pathway |  |  |  |  |  |  |  |  |  |  |  |
|  | Time | 0.843 | 0.463 | [ | -0.064 | , | 1.750 | ] | 3.319 | 0.068 |  |
|  | Group | -0.211 | 1.614 | [ | -3.374 | , | 2.952 | ] | 0.017 | 0.896 |  |
|  | Group*Time | 0.737 | 0.590 | [ | -0.419 | , | 1.893 | ] | 1.560 | 0.212 |  |
| State Hope Scale - Agency |  |  |  |  |  |  |  |  |  |  |  |
|  | Time | -0.195 | 0.406 | [ | -0.990 | , | 0.601 | ] | 0.230 | 0.631 |  |
|  | Group | -1.195 | 1.473 | [ | -4.082 | , | 1.692 | ] | 0.659 | 0.417 |  |
|  | Group*Time | 0.971 | 0.534 | [ | -0.075 | , | 2.017 | ] | 3.311 | 0.069 |  |
| Decisional Conflict Scale - Total |  |  |  |  |  |  |  |  |  |  |  |
|  | Time | -16.911 | 1.419 | [ | -19.692 | , | -14.131 | ] | 142.126 | <0.001 | * |
|  | Group | -10.551 | 5.629 | [ | -21.584 | , | 0.482 | ] | 3.513 | 0.061 |  |
|  | Group*Time | 5.519 | 1.965 | [ | 1.667 | , | 9.370 | ] | 7.885 | 0.005 | * |
| Decisional Conflict Scale - Being informed | | |  |  |  |  |  |  |  |  |  |
|  | Time | -24.705 | 2.731 | [ | -30.057 | , | -19.354 | ] | 81.863 | <0.001 | * |
|  | Group | -16.551 | 9.735 | [ | -35.632 | , | 2.530 | ] | 2.890 | 0.089 |  |
|  | Group*Time | 9.724 | 3.660 | [ | 2.550 | , | 16.898 | ] | 7.058 | 0.008 | * |
| Decisional Conflict Scale - Values clarity | |  |  |  |  |  |  |  |  |  |  |
|  | Time | -25.039 | 3.143 | [ | -31.200 | , | -18.878 | ] | 63.452 | <0.001 | * |
|  | Group | -26.314 | 9.898 | [ | -45.713 | , | -6.915 | ] | 7.068 | 0.008 | * |
|  | Group*Time | 14.237 | 4.072 | [ | 6.256 | , | 22.218 | ] | 12.225 | <0.001 | * |
| Decisional Conflict Scale - Support |  |  |  |  |  |  |  |  |  |  |  |
|  | Time | -10.997 | 1.732 | [ | -14.391 | , | -7.603 | ] | 40.327 | <0.001 | * |
|  | Group | 2.618 | 6.246 | [ | -9.624 | , | 14.860 | ] | 0.176 | 0.675 |  |
|  | Group*Time | 0.519 | 2.533 | [ | -4.446 | , | 5.484 | ] | 0.042 | 0.838 |  |
| Decisional Conflict Scale - Uncertainty | |  |  |  |  |  |  |  |  |  |  |
|  | Time | -14.242 | 2.353 | [ | -18.854 | , | -9.630 | ] | 36.637 | <0.001 | * |
|  | Group | -4.768 | 8.470 | [ | -21.368 | , | 11.832 | ] | 0.317 | 0.573 |  |
|  | Group*Time | 1.187 | 3.265 | [ | -5.212 | , | 7.586 | ] | 0.132 | 0.716 |  |
| Decisional Conflict Scale - Effective decision | |  |  |  |  |  |  |  |  |  |  |
|  | Time | -10.854 | 1.837 | [ | -14.455 | , | -7.252 | ] | 34.891 | <0.001 | * |
|  | Group | -8.029 | 6.305 | [ | -20.387 | , | 4.329 | ] | 1.622 | 0.203 |  |
|  | Group*Time | 2.312 | 2.282 | [ | -2.161 | , | 6.784 | ] | 1.026 | 0.311 |  |
| KDQOL-36 Subscales |  |  |  |  |  |  |  |  |  |  |  |
| KDQOL-36 - SF12 |  |  |  |  |  |  |  |  |  |  |  |
|  |  |  |  |  |  |  |  |  |  |  |  |
| KDQOL-36 (PCS)^a^ | Time | 1.873 | 2.041 | [ | -2.127 | , | 5.874 | ] | 0.843 | 0.359 |  |
|  | Group | -4.677 | 7.828 | [ | -20.020 | , | 10.666 | ] | 0.357 | 0.550 |  |
|  | Group*Time | 3.590 | 3.328 | [ | -2.934 | , | 10.113 | ] | 1.163 | 0.281 |  |
|  |  |  |  |  |  |  |  |  |  |  |  |
| KDQOL-36 (MCS)^b^ | Time | -0.155 | 1.549 | [ | -2.882 | , | 3.192 | ] | 0.010 | 0.920 |  |
|  | Group | -12.575 | 7.096 | [ | -26.483 | , | 1.333 | ] | 3.140 | 0.076 |  |
|  | Group*Time | 6.093 | 2.343 | [ | 1.501 | , | 6.763 | ] | 6.763 | 0.009 | * |
|  |  |  |  |  |  |  |  |  |  |  |  |
| KDQOL-36 - Burden of kidney disease | |  |  |  |  |  |  |  |  |  |  |
|  | Time | -2.811 | 1.631 | [ | -6.008 | , | 0.385 | ] | 2.972 | 0.085 |  |
|  | Group | -8.694 | 8.102 | [ | -24.574 | , | 7.185 | ] | 1.152 | 0.283 |  |
|  | Group*Time | 4.568 | 2.871 | [ | -1.059 | , | 10.194 | ] | 2.532 | 0.112 |  |
| KDQOL-36 - Symptoms & problems |  |  |  |  |  |  |  |  |  |  |  |
|  | Time | 0.660 | 1.035 | [ | -1.368 | , | 2.689 | ] | 0.407 | 0.523 |  |
|  | Group | -7.944 | 4.636 | [ | -17.031 | , | 1.143 | ] | 2.936 | 0.087 |  |
|  | Group*Time | 2.996 | 1.575 | [ | -0.091 | , | 6.084 | ] | 3.617 | 0.057 |  |
| KDQOL-36 - Effects of kidney disease | |  |  |  |  |  |  |  |  |  |  |
|  | Time | -1.470 | 1.201 | [ | -3.823 | , | 0.883 | ] | 1.499 | 0.221 |  |
|  | Group | -7.111 | 4.148 | [ | -15.241 | , | 1.020 | ] | 2.938 | 0.087 |  |
|  | Group*Time | 4.475 | 1.551 | [ | 1.435 | , | 7.516 | ] | 8.324 | 0.004 | * |

KDQOL=Kidney Disease Quality of Life Questionnaire

^#^Age and sex adjusted; SE=standard error; CI=confidence interval; * p < 0.05

Supplementary Table 2. Changes in level of hope, decisional conflict and quality of life at 4- (Time 2), 8- (Time 3) weeks after the intervention.

KDQOL=Kidney Disease Quality of Life Questionnaire. EM Means= Estimated Marginal Means; SE=standard error; CI=confidence interval; ES=effect size.

^a^ Absolute difference and significance level at time 2; ^b^ Absolute difference and significance level at time 3; ^c^ Cohen's d: effect size between control and experimental group; ^d^ Cohen's d within group at Time 2; ^e^ Cohen's d at Time 3.

*p-value < 0.05; **P-value < 0.01; ***P-value < 0.001.

Supplementary Table 3. Regression coefficient estimates of Hope on Decisional Conflict

| Variables | |  | | Adjusted Model^#^ | | | | | | | | | | | | | | | | | | | | | | |
| --- | --- | --- | --- | --- | --- | --- | --- | --- | --- | --- | --- | --- | --- | --- | --- | --- | --- | --- | --- | --- | --- | --- | --- | --- | --- | --- |
|  | |  | | β | SE | | | |  | 95% CI | | | | | | | | | |  | Wald χ^2^ | | | P | |  |
| Age | | | | 0.148 | 0.123 | | | | [ | | -0.092 | | | , | 0.389 | ] | | 1.459 | | | | 0.227 | | | |  |
| Gender | | | | -3.746 | 2.989 | | | | [ | | -9.605 | | | , | 2.112 | ] | | 1.571 | | | | 0.210 | | | |  |
| Decisional Conflict Scale - Total | | | |  |  | | | |  | |  | | |  |  |  | |  | | | |  | | | |  |
|  | | Time | | -13.378 | 1.082 | | | | [ | | -15.498 | | | , | -11.257 | ] | | 152.902 | | | | <0.001 | | | | * |
|  | | Group | | 0.976 | 3.150 | | | | [ | | -5.198 | | | , | 7.150 | ] | | 0.096 | | | | 0.757 | | | |  |
|  | | State Hope Scale - Total | | -0.466 | 0.146 | | | | [ | | -.752 | | | , | -0.181 | ] | | 10.239 | | | | 0.001 | | | | * |
| ^#^Age and sex adjusted; CI=Confidence interval; SE=Standard error | | | | | | | | | | | | | | | | | | | | | | | | | |  |
| * P < 0.05 |  | |  | | |  |  |  | | | |  |  | | | |  | |  | | | |  | |  |  |
